# Preoperative characteristics and intraoperative factors do not correlate with accomplishments of active straight-leg raising, standing up, and walking after primary total knee arthroplasty

**DOI:** 10.1186/s13018-021-02636-7

**Published:** 2021-08-12

**Authors:** Yoshinori Ishii, Hideo Noguchi, Junko Sato, Ikuko Takahashi, Hana Ishii, Ryo Ishii, Kei Ishii, Shin-Ichi Toyabe

**Affiliations:** 1 Ishii Orthopaedic & Rehabilitation Clinic, 1089 Shimo-Oshi, Gyoda, Saitama, 361-0037 Japan; 2 School of Plastic Surgery, Kanazawa Medical University, 1-1 Daigaku Uchinada, Ishikawa, 920-0253 Japan; 3 Shinshu University Hospital, 3-1-1 Asahi Matsumoto, Nagano, 390-8621 Japan; 4 Iwate Prefectural Ninohe Hospital, 38 Horino, Ninohe, Iwate, 028-6193 Japan; 5 Niigata University Crisis Management Office, Niigata University Hospital, Niigata University Graduate School of Medical and Dental Sciences, 1 Asahimachi Dori Niigata, Niigata, 951-8520 Japan

**Keywords:** total knee arthroplasty, active straight leg raising, standing up; walking, early postoperative rehabilitation

## Abstract

**Background:**

The correlations between patient characteristics and early postoperative functional performances after total knee arthroplasty have not been adequately studied. The purpose of this study was to clarify the effects of preoperative characteristics (sex, age, body mass index, American Society of Anesthesiologists grade, hospital for special surgery score) and intraoperative factors (duration of surgery and tourniquet use) on the time required to accomplish active straight-leg-raising, standing up, and walking as the objective performances for the initiation of early postoperative rehabilitation.

**Methods:**

This cross-sectional retrospective study included 307 patients (384 primary total knee arthroplasties). Postoperative times required until each activity was accomplished were measured. Various preoperative characteristics and intraoperative factors that might affect three objective performances were evaluated.

**Results:**

The postoperative times required before each activity was accomplished were 1.5 ± 0.5 days for active straight-leg-raising, 1.2 ± 0.5 days for standing up, and 1.4 ± 0.7 days for walking. There were no significant correlations between any factor (age, body mass index, hospital for special surgery score, duration of surgery, and tourniquet use) and the three objective performances using Spearman’s correlation coefficient. There were no differences in sex or American Society of Anesthesiologists grade for three objective functional assessments by Wilcoxon rank sum test.

**Conclusions:**

Differences in patient preoperative characteristics and intraoperative factors are unlikely to affect three objective functional performances in the early postoperative period. Therefore, there is no need to consider differences between patients when initiating early postoperative rehabilitation.

## Introduction

The early initiation of postoperative rehabilitation after total knee arthroplasty (TKA) is recommended because it might reduce the length of hospital stay [1] and prevent deep vein thrombosis [2]. To promote early postoperative rehabilitation, surgeons have verified the effectiveness of surgical approaches such as the less invasive capsulotomy procedure [3–5] or without the use of a tourniquet [6] during TKA surgery. In addition, different perioperative anesthetics have been studied to determine their effectiveness at controlling early postoperative pain [7–9]. Several tests have been used to objectively evaluate these outcomes including various functional performances such as the time required to accomplish active straight leg raising (ASLR) [3, 4, 6, 8, 10], standing up [5, 10], and walking [4, 8, 10], the number of walking steps, distance, or speed [8], the Timed “Up & Go” test [3, 8], and the acquired range of motion (ROM) [3, 4, 6, 8].

Because surgical populations are heterogeneous with regards to patients [11] and surgeons [12], it is important for surgeons to understand the effect of different preoperative patient characteristics such as unmodifiable factors (sex, age) and modifiable factors (body mass index (BMI), American Society of Anesthesiologists (ASA) grade [13], Hospital for Special Surgery (HSS) knee score [14]) on early postoperative rehabilitation in accordance with evidence-based approaches. However, early rehabilitation practices vary widely according to clinical reports [1, 2, 5]. To date, no studies have analyzed the effects of preoperative patient characteristics or intraoperative factors such as duration of surgery and tourniquet use, the prolongation of which is a negative factor for clinical outcomes after TKA [15, 16], on the initiation of early rehabilitation.

Therefore, the purpose of this study was to clarify the effects of preoperative patient characteristics (sex, age, BMI, ASA grade [13], HSS knee score [14]) and intraoperative factors (duration of surgery and tourniquet use) on the time required to accomplish ASLR, standing up, and walking as objective performances for the initiation of early postoperative rehabilitation.

## Materials and methods

Our institutional review board approved this cross-sectional retrospective study of medical records and the analysis of pertinent data from patients treated with primary hybrid TKA (cemented tibia, uncemented femur, no patellar replacement) from October 2004 to June 2020. Overall, 307 patients (384 TKAs) were included in this study. Informed consent was waived by the institutional review board because of the retrospective study design. The inclusion criterion was a diagnosis of primary osteoarthritis. Exclusion criteria included previous revision arthroplasty or tibial osteotomy, or a history of rheumatoid arthritis. The clinical characteristics of the patients are summarized in Table 1.

**Table 1:** Patient demographics

Characteristics (*N* = 307 patients, 384 knees)	Mean (SD)
Age at the first TKA	73 (8)
Sex (male/female; patients, knees)	53/254, 63/321
Body height (cm)	151 (7)
Body weight (kg)	60 (11)
BMI (kg/m^2^)	26 (4)
Preoperative HSS score	44 (11)
ASA* Grade (I/II) (knees)	54/330
Operation time (min.), ≥90 min.(knees)	57 (10), range 36–116, 5
Tourniquet time (min.), ≥90 min.(knees)	60 (10), range 38–118, 6
Active SLR (postoperative days)	1.5 (0.5), range 1–3
Standing up (postoperative days)	1.2 (0.5), range 1–5
Walking (postoperative days)	1.4 (0.7), range 1–5

SD, standard deviation; TKA, total knee arthroplasty; BMI, body mass index; HSS score, Hospital for Special Surgery score [14], SLR, straight leg raising, ASA* [13], American Society of Anesthesiologists.

### Surgical procedure and rehabilitation protocol

The LCS® Total Knee System (DePuy, Warsaw, IN, USA) was used on all patients, with either a posterior cruciate ligament (PCL)-retaining design or a PCL-substituting design. All TKAs were performed using a unilateral procedure under general anesthesia without using adductor canal block and femoral nerve block; all patients with bilateral disease were scheduled to undergo staged bilateral TKA. An MT-720 tourniquet system (Mizuho-Ika, Tokyo, Japan) that adjusts its pressure in synchrony with the systolic blood pressure (SBP), was used to prevent excessive intraoperative hemorrhage. The tourniquet was initially inflated based on the SBP recorded before the skin incision. This maintained a pressure of 100 mmHg greater than the intraoperative SBP of the patient. The tourniquet was released after wound closure in all cases.

**Fig. 1:**
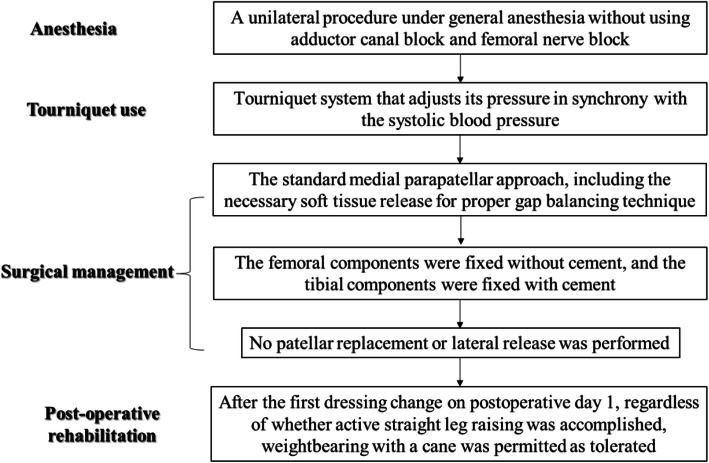
A flow-chart of the enhanced recover after surgery protocol for all 384 knees in this study

One senior surgeon performed all TKAs using a standardized technique with the standard medial parapatellar approach, including the necessary soft tissue release for proper gap balancing *with mechanical alignment procedure*. An anterior midline skin incision was made, extending from the level of the distal tibial tubercle to approximately 6 cm proximal to the superior border of the patella. In all knees, the femoral components were fixed without cement, and the tibial components were fixed with cement. No patellar replacement or lateral release was performed in any case. All wounds were closed in the same manner by one surgeon. Capsular repair was performed with an approximately 2-cm interval. A bulky compression dressing was applied. After the first dressing change on postoperative day 1, regardless of whether ASLR was accomplished, weightbearing with a cane was permitted as tolerated under the supervision of a therapist, and additional exercises were allowed despite the accomplishment of ASLR as detailed below. Passive ROM exercises were performed daily beginning at postoperative week 1. Patients received at least 2 h of daily physical therapy that consisted of isometric exercises, passive ROM, active assisted ROM, quadricep and hamstring strengthening, and gait training including ascending and descending stairs (Fig. 1).

### Evaluation of ASLR, standing up, and walking, and their affecting factors

ASLR was evaluated after each patient’s first dressing change on postoperative day 1 (approximately 15 h postoperatively). ASLR was performed in the supine position with a straight leg on a bed. The examiner judged ASLR as accomplished when the patient’s heel on the TKA-side was raised off the bed, regardless of the height and velocity of raising. Next, standing up evaluated from a sitting position in a chair. The examiner judged standing up as accomplished when the patient’s heel on the TKA-side touched the floor and a load could be applied, regardless of the use of assistive devices. Finally, walking was performed using parallel bars. A patient who could walk more than one round trip within a 5 m parallel bar was defined as accomplished. The respective postoperative periods (in days) required until the achievement of ASLR, standing up, and walking were determined. Thus, if patients did not accomplish these tasks on the first day, the next earliest confirmation attempt was the second day. Therefore, the timescale for performance did not have a granularity of one hour but one day. Various factors that might have impacted on the accomplishment of these performances immediately after TKA were evaluated. First, patient characteristics (*unmodifiable patient factors:* sex, age; *modifiable patient factors:* BMI, ASA grade [13], preoperative HSS knee score [14]) were investigated as preoperative factors. Second, the duration of surgery and tourniquet use were evaluated as intraoperative factors because their prolongation was regarded as a negative factor for clinical outcomes after TKA [15, 16].

Postoperative analgesia using a combination of diclofenac sodium (50 mg; suppository), ketoprofen (15 mg; intramuscular injection), and pentazocine (15 mg; intramuscular injection), according to each patient’s request during the early postoperative period was described previously [17].

### Statistical analysis

Spearman’s rank correlation coefficient was used to investigate the association between any two variables (Table 2). The Wilcoxon rank sum test was used to determine differences between two groups. The strength of the correlation of the rank coefficients was defined as: strong = 0.70–1.0, moderate = 0.40–0.69, or weak = 0.20–0.39. In all tests, a *p-*value less than 0.05 was considered significant. Post hoc power analyses were performed after the study. The power of Spearman’s rank correlation test with a medium effect size (0.3) and α-error of 0.05 was 0.999, and the power of the Wilcoxon rank sum test was a medium effect size (0.29) and α-error of 0.05 was 0.533. All statistical analyses were performed using IBM SPSS Statistics version 23 (IBM Japan, Tokyo, Japan). Values are expressed as the mean ± standard deviation.

**Table 2:** Univariate analyses using Spearman’s correlation coefficient for continuous variables and Wilcoxon rank sum test* for discrete variables

Performances		Gender*	Age	BMI	ASA*	HSS	Operation. Time	Tourniquet Time
ASLR	R		-0.022	0.009		0.048	-0.056	-0.016
	Sig.	0.397	0.669	0.862	0.995	0.343	0.273	0.758
Standing up	R		0.031	-0.002		0.025	0.045	0.052
	Sig.	0.714	0.545	0.966	0.084	0.620	0.379	0.309
Walking	R		0.029	-0.005		0.025	0.095	0.086
	Sig.	0.536	0.566	0.923	0.170	0.632	0.064	0.092

BMI, body mass index; ASA grade [13]*, American Society of Anesthesiologists ; HSS knee score, Hospital for Special Surgery score [14].

## Results

The postoperative times required before each activity was accomplished were 1.5 ± 0.5 days for ASLR, 1.2 ± 0.5 days for standing up, and 1.4 ± 0.7 days for walking. All patients achieved ASLR, standing up, and walking by postoperative day 3, 5, and 5, respectively (Table 1). In addition, 54.2% (208/384) of patients accomplished ASLR, 80.2% (308/384) were capable of standing up, and 70.6% (271/384) were capable of walking which constituted the three objective performances on postoperative day 1."

Univariate analyses using Spearman’s correlation coefficient for continuous variables and discrete variables indicated no significant correlations between any factors (age, BMI, HSS knee score [14], duration of surgery, and tourniquet use) and ASLR, standing up, and walking (Table 2). In addition, there were no differences between discrete variables such as sex (male vs female) and ASA grade [13] (I vs II) using the Wilcoxon rank sum test with regards to the three functional objective assessments (Table 2).

## Discussion

The important finding of this study was that preoperative characteristics and intraoperative factors among patients did not correlate with the three objective performances including ASLR, standing up, and walking after TKA surgery. Thus, this study indicated that the initiation of early postoperative rehabilitation could be promoted without considering unmodifiable preoperative factors such as age and gender, or modifiable preoperative factors such as preoperative BMI, ASA grade [13], and HSS knee score [14], or intraoperative factors such as duration of surgery and tourniquet application.

Regarding the three objective performances in this study, ASLR was reported to assess the recovery of quadricep function after TKA [3, 4, 10]. In addition, standing up from a chair is an important test that focuses on the extensor mechanism of the knee and shows the contracting ability of the quadricep femoris muscle [18], and which are often reported after TKA with clinical [19] and biomechanical analysis [20]. Finally, walking and factors related to walking such as number of steps, distance, or speed are commonly evaluated as landmarks of the recovery of functional performance after TKA [8] in addition to whether patients could walk independently of an assistive device. The observations related to standing up and walking after TKA might be more realistic and authentic markers for the initiation of early postoperative rehabilitation compared with ASLR testing, which is performed with the patient in a supine position. We think that these functional assessments have advantages over other performance tests such as measuring the number of walking steps, distance, or speed, Timed “Up & Go” test, and the acquired ROM because it is easy for the observer to judge the accomplishment of each test and they are less of a burden for the patient during the early postoperative period.

The time to accomplish each performance was comparable to those reported previously. Most of the mean times for ASLR in previous reports were within 2 days, including 1.92 days by Bourke et al. [3], 1.1 days by Li et al. [4], 4.6–6.4 h by Zhou et al. [6], and (median) 1.5 days by Ishii et al. [10]. Furthermore, previous reports indicated accomplishments within 3 days for standing up, including mean 2.6 days by Lisi et al. [5], 8.56–8.60 h by Shah et al. [8], and (median) 1.3 days by Ishii et al. [10]. Finally, the mean time to accomplish walking was within 3 days, including 1.7 days by Li et al. [4], 2.9 days by Lisi et al. [5], and (median) 1.4 days by Ishii et al. [10] despite using an assistive device. However, considering that the non-accomplishment rates for postoperative day 1 were 45.8% for ASLR, 19.8% for standing up, and 29.4% for walking, the introduction of an enhanced recovery procedure as recently reported [1] may help to improve these outcomes.

Whether there is a correlation between clinical outcomes and unmodifiable patient factors such as age and sex in TKA patients remains inconclusive. Some studies reported that older people have worse clinical outcomes [15, 21] whereas others reported older people can expect outcomes equivalent to those in younger people (over 80 vs less than 80 years [22], over 90 vs less than 90 years [23]). Regarding sex differences, some [24, 25] studies reported that men had better outcomes, whereas other studies [21, 26] reported better outcomes in women. However, the present study indicated that no factors affected the accomplishments of the three objective performances. We speculate that the period for assessment testing might not be sufficient to reflect the differences of aging and sex because the longest follow up was only 3 days for ASLR and 5 days for standing up or walking after TKA surgery. Conversely, other studies evaluated these outcomes between the 30-day readmission rate in a short-term follow up study [15, 21, 22] and a long-term follow up study [26], which might be sufficiently long to detect or reveal the characteristics of aging and sex.

Regarding modifiable patient factors, most reports concluded that the better the preoperative scores, the better the postoperative outcomes such as clinical score [24, 25], BMI [15, 27], and ASA grade [15, 22]. Because these factors are modifiable, strategies focused at reducing obesity and treating comorbid conditions prior to TKA might improve functional outcomes following TKA. In particular, the BMI can be improved relatively easily compared with the other two factors. Most reports indicated that a BMI less than 29–30 is favorable, in order to decrease the risk of most TKA postoperative complications [27]. It is desirable to improve these modifiable factors before proceeding to TKA surgery [27]. However, the current study indicated that these three factors did not affect the initiation of early rehabilitation. Two reasons may contribute to this outcome. One is that all patients were ASA grade I or II and had a general condition did not prevent early rehabilitation. TKA surgery for patients with ASA grade III or higher requires special attention because ASA grades are an important predictor of the development of postoperative complications [15]. Because all patients included in the present study were only ASA grade I or II, no patients suffered from debilitating medical conditions that substantially increased their risk of serious perioperative complications or death. Another factor is that the BMI of all patients was less than 40 kg/m^2^, which is the cut-off value for the increased incidence of complications following primary TKA [15]. Even considering these favorable conditional factors, this study suggests that a personalized rehabilitation program based on patient characteristics may not be required when promoting early postoperative rehabilitation.

Regarding intraoperative patient factors such as duration of surgery and tourniquet application, the prolongation of these times is likely to be a disadvantage for early rehabilitation. Belmont et al. [15] reported that increased operative time impacted the development of postoperative complications. Because all surgeries were performed by one surgeon with the same procedure in this study, there was no inter-surgeon bias. Longer surgery was needed when the degree of deformation and contracture of the osteoarthritic knee of the patient was regarded as severe. Therefore, the use of increasingly invasive procedures, which are likely to delay early rehabilitation, were often undertaken to adequately correct severe issues. Thus, the operation time was used as a proxy measurement of the difficulty of surgery in this study. Cases where the tourniquet time was longer had increased damage related to the compression of the thigh muscle. Clinical study reported a positive correlation between the degree of neuromuscular injury and the duration of tourniquet application [16]. Thus, it is reasonable to assume delayed early rehabilitation in cases with longer times, but no significant correlations were demonstrated in this study. However, there were only five cases (1.3%) of surgery ≥90 min and six knees (1.6%) requiring tourniquets for ≥90 min. A previous study reported that an operative time of > 135 minutes was one of the most important risk factors for the development of postoperative complications [15]. In the current study, few patients underwent highly invasive procedures that might indicate negative effects on the operated knee. Therefore, the current study suggests that intraoperative factors have no significant effect on the initiation of early rehabilitation, unless extremely long durations of surgery or tourniquet use are required.

This study had several limitations. First, this was a retrospective medical record and database review study, which has inherent limitations. *Second, this study involved the patients who underwent the same procedure such as mechanical alignment approach with gap balancing technique. Thus, the results may not be applicable to other procedures, such as kinematic alignment approach with measured resection technique. Third*, we did not use multimodal analgesics such as peripheral nerve blocks or periarticular infiltration analgesia, which are commonly used for postoperative pain control following current TKA surgery. However, multimodal analgesic techniques are associated with certain limitations such as an increased risk of falls, delayed rehabilitation after nerve block [9], and disparities in the results related to infiltration techniques in periarticular infiltration analgesia [7]. Therefore, we recognized such procedures as inappropriate to accomplish the purpose of this study. *Forth*, subjective determinants such as postoperative pain were not evaluated, although poorly-controlled acute postoperative pain can potentially delay postoperative rehabilitation [28]. However, the main purpose of this study was not to clarify the effects of a patient’s individual threshold of pain tolerance, which is a subjective determinant, but rather the effects of preoperative characteristics and intraoperative factors on three *objective* performances. Thus, subjective assessments were excluded from the current study. Finally, the three objective performances as indicators of starting early rehabilitation were not examined on the day of TKA surgery; therefore, future clinical studies are needed to demonstrate their use.

Despite these limitations, the strength of this study is that surgeon-related bias was not included because all the surgeries were completed by a single experienced surgeon using current designs, and who used the same procedure and similar forms of instrumentation for all patients. In addition, the present study identified a correlation between preoperative characteristics and intraoperative factors, and three functional objective performances as an evidence-based approach for the initiation of early postoperative rehabilitation after TKA, which is in accord with a consensus statement issued by the NIH in 2003 identifying the need for rehabilitation after TKA [29]. Considering the current findings, surgeons do not need to stratify patients based on their preoperative characteristics and intraoperative factors, and therefore might better inform patients regarding the expected outcomes, thus reducing the gap between patient expectations and outcomes. Finally, the results of this study may be useful for the planning of early rehabilitation after TKA surgery.

## Conclusion

In conclusion, differences in preoperative characteristics and intraoperative factors among patients are unlikely to affect three objective functional performances during the early postoperative period. Therefore, patient differences do not need to be considered when promoting early postoperative rehabilitation. The present study also supports the effectiveness of unsupervised home exercise as a rehabilitation strategy after primary TKA, which was recently reported to be non-inferior to formal individualized supervised outpatient physical therapy [30], even in the early postoperative period when appropriately-selected patients received adequate clinical support.

## Data Availability

The datasets used and/or analyzed during the current study are available from the corresponding author on reasonable request.
